# Epigenetic modifications promote the expression of the orphan nuclear receptor NR0B1 in human lung adenocarcinoma cells

**DOI:** 10.18632/oncotarget.9012

**Published:** 2016-04-26

**Authors:** Yongjie Lu, Yunqiang Liu, Shunyao Liao, Wenling Tu, Ying Shen, Yuanlong Yan, Dachang Tao, Yilu Lu, Yongxin Ma, Yuan Yang, Sizhong Zhang

**Affiliations:** ^1^ Department of Medical Genetics and Division of Human Morbid Genomics, State Key Laboratory of Biotherapy, West China Hospital, West China Medical School, Sichuan University, Chengdu, Sichuan Province, China; ^2^ Diabetic Center and Institute of Transplantation, Sichuan Academy of Medical Science & Sichuan Provincial People's Hospital, School of Medicine, University of Electronic Science and Technology of China, Chengdu, Sichuan Province, China

**Keywords:** NR0B1, lung adenocarcinoma, DNA methylation, histone modifications, cell self-renewal

## Abstract

The ectopic activation of *NR0B1* is involved in the development of some cancers. However, the regulatory mechanisms controlling *NR0B1* expression are not well understood. Therefore, the epigenetic modifications promoting *NR0B1* activation were examined in this study. NR0B1 protein was detected in cancerous tissues of more than 50% of human lung adenocarcinoma (ADCA) cases and tended to be expressed in low-differentiated cancerous tissues obtained from males. Nevertheless, NR0B1 activation in ADCA has not previously been correlated with DNA demethylation. NR0B1 expression was not detected in 293T cells, although it contains a hypomethylated *NR0B1* promoter. Treating 293T cells with a histone deacetylase inhibitor increased acetylated histone H4 binding to the *NR0B1* promoter and activated *NR0B1* expression. In contrast, treatment with histone methylase inhibitors decreased the methylation of histones H3K9 and H3K27 and slightly induced *NR0B1* transcription. Furthermore, the level of acetyl-histone H4 binding to the *NR0B1* promoter increased, whereas the occupancy of H3K27me3 was lower in cancerous tissues than in non-cancerous tissues. Similar histone occupancies were confirmed in a comparison of cancerous tissues with strong, moderate and negative *NR0B1* expression. In conclusion, this study shows that CpG methylation within the *NR0B1* promoter is not involved in the *in vivo* regulation of *NR0B1* expression, whereas the hyperacetylation of histone H4 and the unmethylation of histones H3K9 and H3K27, and their binding to the *NR0B1* promoter results in decondensed euchromatin for *NR0B1* activation.

## INTRODUCTION

Nuclear receptor subfamily 0 group B member 1 (*NR0B1*), which is located on the X chromosome, is abundantly expressed in normal reproductive testicular and ovarian tissues. The precise levels and timing of *NR0B1* gene expression are critical for gonadal differentiation and sex determination during embryogenesis [1]. In addition, *NR0B1* is ectopically activated in several types of cancers, including endometrial carcinoma [2], ovarian carcinoma [3], prostate carcinoma [4], Ewing's sarcoma [5, 6], lung adenocarcinoma (ADCA) [7], breast cancer [8] and hepatocellular carcinoma [9]. Thus, *NR0B1* is regarded as a typical X-linked cancer/germline gene (CG-X).

To date, the expression of CG-X genes has commonly been thought to be controlled by epigenetic modifications, especially associated with the demethylation of critical CpG residues within their promoter regions [10]. Is the activation of the *NR0B1* gene in cancerous tissues under the control of active DNA demethylation? Oda *et al* reported that the expression level of *NR0B1* was inversely correlated with the proportion of methylated CpG sites within the *NR0B1* promoter in ADCA [7], suggesting that DNA methylation is involved in the activation of *NR0B1* in ADCA. However, this correlation was not observed in our specimens. The CpG sites within the *NR0B1* gene promoter were almost unmethylated in the *in vivo* tissues and cells obtained from males, independent of *NR0B1* expression status. This preliminary result indicates that the hypomethylation of CpG sites within the *NR0B1* gene promoter is not sufficient to trigger *NR0B1* expression in ADCA. Hence, it is not yet known what causes the activation of *NR0B1* in ADCA.

To address this issue, we thoroughly investigated the epigenetic modifications, including DNA methylation and histone modifications, within the *NR0B1* promoter region that regulates its gene expression in clinical ADCA samples and cultured cells. Furthermore, based on the level of *NR0B1* expression in ADCA cells with different clinical stages, our results indicate that epigenetic modifications promote *NR0B1* activation to maintain the self-renewal of cancer cells.

## RESULTS

### NR0B1 expression tends to be activated in male ADCA tissues with low differentiation

**Figure 1:**
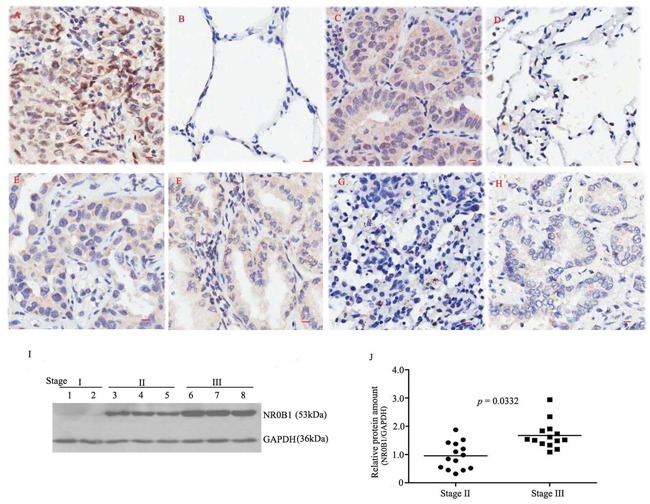
Expression profile of the *NR0B1* protein in human lung adenocarcinoma samples **A.** An example showing strong NR0B1-positive staining in cancerous tissue from a male case of clinical stage III cancer but not in the adjacent non-cancerous tissue **B, C.** An example showing strong NR0B1-positive staining in cancerous tissue from a female case of clinical stage III cancer but not in the adjacent non-cancerous tissue **D, E-F.** An example showing a moderate immunoreactive signal for NR0B1 in the cancerous tissues from one male case (E) and one female case (F) of clinical stage II cancer. **G-H.** Representative image showing NR0B1-negative staining in cancerous tissues (from one female case in stage III (G) and one male case in stage I cancer (H)). Scale bar = 10 μm. **I.** Varying amounts of NR0B1 protein were detected in different male cancerous tissues that were obtained from several representative cases of stages I, II and III cancer and analyzed using immunoblotting. **J.** Relative NR0B1 protein levels (versus GAPDH) in male cancerous tissues in stage II and stage III (14 cases/stage). The results showed that the NR0B1 protein was present at a higher level in the male stage III cancerous tissues than the male stage II cancerous tissues.

The ectopic activation of NR0B1 was investigated in 160 ADCA cases using IHC analysis. The NR0B1 signal was present in 87 cases (54.37% of a total of 160 cases, Table 1) and was detected in both the nucleus and the cytoplasm of ADCA cells but not in the paired adjacent noncancerous lung cells (Figure 1A–1F). Notably, the NR0B1 protein was expressed more frequently in males (53 of 83 cases, 63.86%) than in females (34 of 77 cases, 44.16%), with a *p* value of 0.0124 (Table 1). Moreover, a strong NR0B1 signal was also more frequently present in males than in females (*p*=0.0070, Table 1).

**Table 1:** NR0B1 expression profile in the human lung adenocarcinoma samples with different clinical stage

		No. of Cases with different NR0B1 signal level			*p* value ^a^
		Strong (++)	Moderate (+)	Negative (−)	
**Gender**	**Male**	30	23	30	0.0124^b^*
	**Female**	15	19	43	0.0070^c^**
**Clinical Stage**	**I**	0	0	5(2)	0.0925^e^
	**II**	28(19)^d^	32(18)^d^	55(25)^d^	0.0492^f^*
	**III**	17(11)^d^	10(5)^d^	13(3)^d^	0.9366^g^

a Pearson's chi-squared test is applied in the calculation.

* *p* value < 0.05,

** *p* value < 0.01

b,c The *p* value is calculated by comparing the number of male and female cases^b^ between NR0B1-positive group (++ and +) and -negative group (−) and ^c^ between NR0B1-strong group (++) and -negative group.

d The number in the parentheses is that of male cases.

e,g The *p* value is calculated by comparing the number of ^e^ both male and female cases, ^f^ only male cases, and ^g^only female cases, of the stage II and III between NR0B1-positive group and negative group.

In addition, the NR0B1 signal was observed in approximately one-half of the specimens obtained from clinical stage II and two-thirds of the specimens obtained from stage III patients, but it was not detected in any of the 5 stage I patients (Figure 1G–1H, Table 1). In particular, the NR0B1 signal was significantly stronger in the tissues obtained from male cases of stage III than in those obtained from male stage II patients, whereas no difference was observed in NR0B1 expression between clinical stages in females (*p*=0.049 for males, *p*=0.937 for females, Table 1). Furthermore, immunoblotting analysis of whole protein that were extracted from male cancerous tissues at stages II and III confirmed that the NR0B1 protein was expressed at higher levels in the poorly differentiated cases that were stage III (Figure 1I–1J).

### NR0B1 expression in ADCA does not result from active DNA demethylation in the CpG island (CGI) region of the *NR0B1* gene

**Figure 2:**
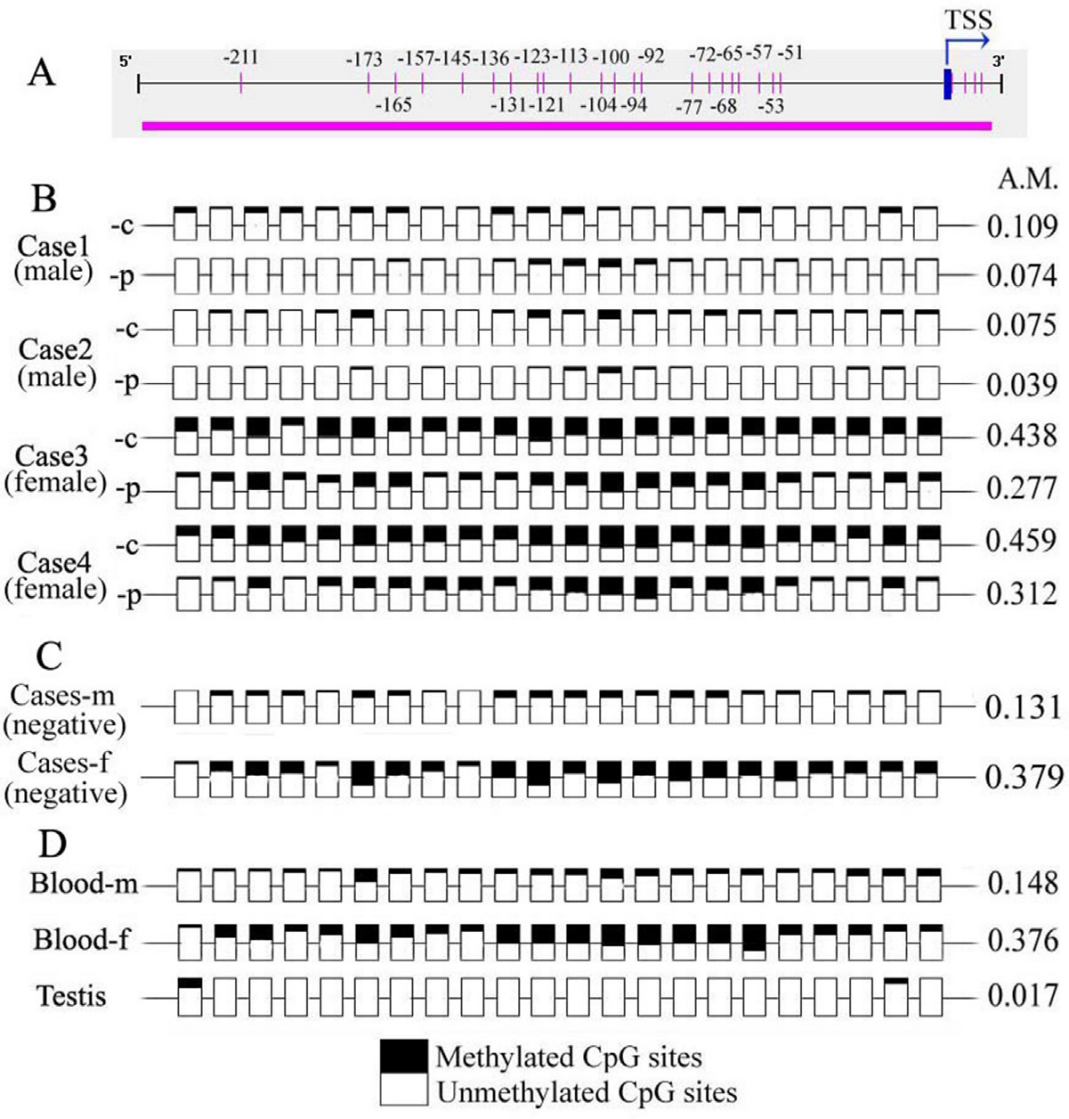
Methylation status of CpG sites within the *NR0B1* promoter in human samples **A.** Scheme showing the CpG sites within the *NR0B1* promoter. The numbers indicate the positions of the CpG sites, with the transcription start site (TSS) considering to be +1. **B.** The pattern of methylation of the *NR0B1* CpGs in cancerous (−c) and normal non-cancerous (−p) tissues in male and female lung adenocarcinoma cases. Cases 1 and 3: Clinical stage III with strong NR0B1 expression; and cases 2 and 4: stage II with moderate NR0B1 expression. **C.** Cases (negative) showing a mixture of cancerous tissues with a negative NR0B1 signal from 10 male (−m) and female (−f) cases, respectively. **D.** The *NR0B1* methylation status is shown for the genomic DNA from male (−m) and female (−f) peripheral blood and testis samples. Empty squares indicate unmethylated cytosine residues, and shaded squares indicate methylated cytosine residues. A.M.: average ratio of methylated CpG sites.

The methylation pattern of the *NR0B1* CGI was investigated in cancerous and paired adjacent noncancerous tissues using 10 cases of ADCA, including 5 males and 5 females. Bisulfite sequencing revealed that almost all of the CpG sites were unmethylated in both the cancerous and the noncancerous tissues obtained from male cases, and more than 50% of the CpG sites were unmethylated in those obtained from female cases. In particular, no significant difference was observed in the methylated CpG level between cancerous and noncancerous tissues (Figure 2A–2B). Furthermore, the hypomethylated status of *NR0B1* CGI was confirmed in the NR0B1-negative cancerous tissues that were obtained from another 10 male and female cases (Figure 2C).

Additionally, the methylation pattern of the *NR0B1* CGI was investigated in normal tissues and cells from males and females. The peripheral blood from 10 healthy subjects without *NR0B1* transcription and the testis tissues of 2 obstructive azoospermic patients with normal spermatogenesis and *NR0B1* transcription were examined. Similarly, the CpG sites were not frequently methylated in male blood and testis, and less than half of the CpG sites were methylated in female blood (Figure 2D). Given that females have two copies of the *NR0B1* gene, the increased methylation level in females may reflect X inactivation.

Due to the limitations associated with using human samples, the relationship between DNA methylation and *NR0B1* expression could not be verified in multiple human tissues. Therefore, the methylation status of the homologous *Nr0b1* CGI was examined in mouse tissues and cells using pyrosequencing analysis. The results also indicated that the CpG sites in the *Nr0b1* gene were hypomethylated (methylation rate: 0~30%) in the heart, liver, brain, lungs, pancreas and peripheral leukocytes of male mice, and the average methylation ratio was also less than 40% in the parallel tissues and cells of female mice (Supplementary Figure S1). Similarly, the methylation level of the *Nr0b1* CGI was not different between testis tissues with *Nr0b1* expression and other tissues without *Nr0b1* expression in male mice or between ovarian tissues and other tissues in female mice (Supplementary Figure S1-S2). Furthermore, the methylation level of the CpG sites in the *Nr0b1* promoter did not obviously change as the expression of *Nr0b1* shifted during embryogenesis and testis development (Supplementary Figure S1-S2). These results in mice are in agreement with those obtained in humans, indicating that DNA methylation is not involved in the regulation of *NR0B1/Nr0b*1 gene expression *in vivo*.

### Hypermethylated *NR0B1* CGI silences its expression *in vitro*

**Figure 3:**
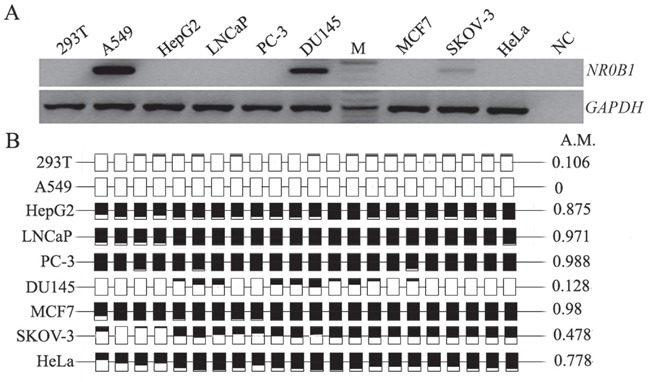
Correlation analysis of *NR0B1* expression with its CGI methylation status in nine cell lines **A.** The *NR0B1* mRNA was detected using RT-PCR in A549, DU145 and SKOV-3 cells but not in 293T, HepG2, LNCaP, PC-3, MCF7 and HeLa cells. NC: Water acted as a negative control for the RT-PCR template. **B.** Relative methylated levels of the *NR0B1* CGIs in different cells. M: DNA ladder. A.M.: average ratio of methylated CpG sites.

**Figure 4:**
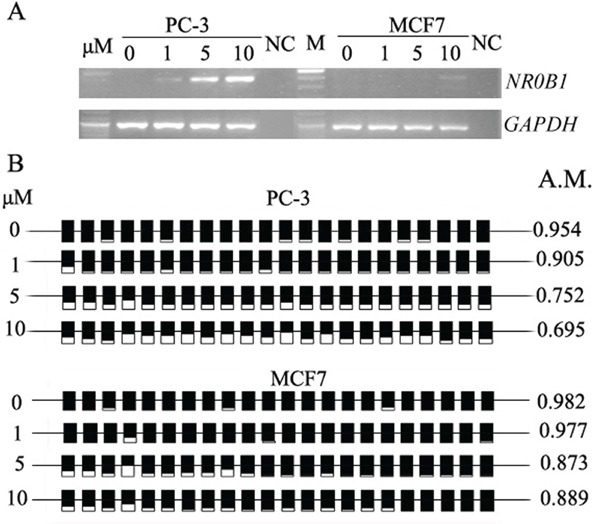
DNA demethylation activates *NR0B1* expression **A.** The expression of *NR0B1* was activated in PC-3 and MCF7 cells that were treated with the DNA methyltransferase inhibitor AZA. NC: Water acted as a negative control for the RT-PCR template. **B.** *NR0B1* CGI was partially demethylated in cells that were treated with AZA. A.M.: average ratio of methylated CpG sites.

Despite the fact that DNA methylation was not correlated with *in vivo NR0B1* gene expression, a relationship was observed between *NR0B1* expression and CGI methylation status in several cultured cells. For example, *NR0B1* was expressed in A549, DU145 and SKOV-3 cells, all of which contain a hypomethylated *NR0B1* CGI, whereas *NR0B1* was not present in HepG2, LNCaP, PC-3, MCF7 and HeLa cells, which contain a hypermethylated *NR0B1* CGI (Figure 3). Meanwhile, the expression of *NR0B1* in PC-3 and MCF7 cells and mouse *Nr0b1* in GC-1 cells was activated by treating the cells with the DNA methyltransferase inhibitor AZA (Figure 4A, Supplementary Figure S3A). Subsequent bisulfite sequencing and pyrosequencing confirmed that the *NR0B1/Nr0b1* CGI was demethylated in PC-3, MCF7 and GC-1 cells after AZA treatment (Figure 4B, Supplementary Figure S3B).

To further examine the effect of the methylation status of the *NR0B1/Nr0b1* CGI on the regulation of gene expression *in vitro*, the 5′ upstream sequences of *NR0B1* and its mouse homolog *Nr0b1* were sub-cloned into a CpG-free luciferase reporter vector. After *in vitro* methylation treatment with CpG methyltransferase, the luciferase activity of the methylated *NR0B1* and *Nr0b1* constructs was clearly repressed (Supplementary Figure S4A-S4B), in agreement with the results described in a previous report [7]. These results demonstrate that DNA methylation is involved in the regulation of *NR0B1/Nr0b1* expression *in vitro* and that hypermethylating the *NR0B1* CGI silences the expression of this gene in cultured cells.

### Acetyl-histone H4 binding to the *NR0B1* gene promoter activates its expression

**Figure 5:**
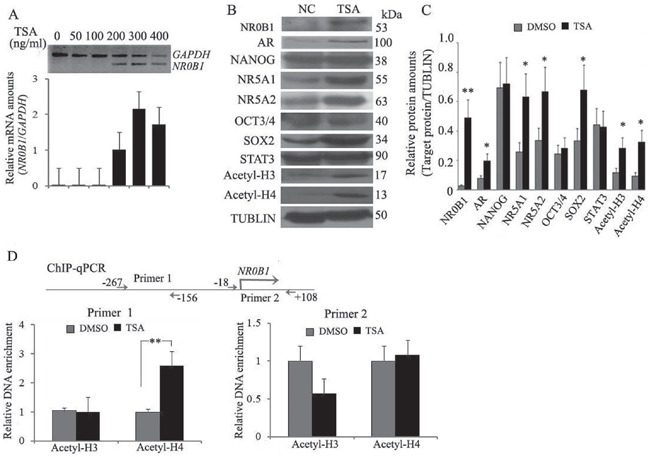
Histone H4 acetylation promotes *NR0B1* gene expression **A.** The expression of NR0B1 mRNA was activated in 293T cells that were treated with the HDAC inhibitor TSA (upper panel: gel electrophoresis results of RT-PCR; low panel: qRT-PCR results). **B.** Protein levels of acetyl-histone H3 and H4 and the transcription factors AR, NANOG, NR5A1, NR5A2, OCT3/4 and SOX2 were compared before and after TSA treatment in 293T cells. TUBULIN acted as an internal control. **C.** Relative protein amounts (versus TUBULIN) of NR0B1, acetyl-histone H3 and H4, AR, NANOG, NR5A1, NR5A2, OCT3/4 and SOX2 in 293T cells before and after TSA treatment. **D.** ChIP-qPCR analysis of the *NR0B1* promoter region using anti-acetyl-histone H3 and H4 antibodies in 293T cells before and after TSA treatment. The promoter region from −267 to −156 of the *NR0B1* gene was significantly enriched in TSA-treated 293T cells, as detected using anti-acetyl-histone H4 antibodies. * *p* value <0.05 and ** *p* value <0.01 in the Student's *t*-test.

While investigating the correlation between *NR0B1* expression and its promoter CGI methylation in cultured cells, we unexpectedly found that the transcription of the *NR0B1* gene was almost undetectable in 293T cells, even though the *NR0B1* CGI was slightly methylated (Figure 3A–3B). The features of the *NR0B1* gene were similar in 293T cells to those that were observed in male tissues and cells *in vivo*. Thus, 293T cells were selected to explore the effect of other epigenetic factors, such as histone modifications, on the expression of the *NR0B1* gene. To evaluate the contribution of histone acetylation to the epigenetic regulation of the *NR0B1* gene, the common histone deacetylase (HDAC) inhibitor TSA was used to treat 293T cells. Interestingly, the expression of the *NR0B1* gene was activated after treatment with TSA, and the up-regulation of *NR0B1* mRNA occurred in a dose-dependent manner, with maximal induction observed at a TSA dose of 300 ng/mL (Figure 5A).

Considering that the expression of *NR0B1* is under the control of a cross-talking network that involves transcriptional factors such as NANOG, OCT3/4, androgen receptor (AR), NR5A1 (also named steroidogenic factor 1), NR5A2 (also named liver receptor homolog-1), SOX2 and STAT3 during embryogenesis in addition to the production of steroid hormones [11–15], we next analyzed changes in these factors in 293T cells that were treated with TSA. As shown in Figure 5B and 5C, the expression levels of AR, NR5A1, NR5A2 and SOX2 were significantly increased after treatment. Because these four trans-acting factors can promote *NR0B1* expression, we investigated whether *NR0B1* activation resulted from an enrichment of related trans-acting factor(s) in TSA-treated 293T cells. To address this question, expression vectors containing AR, NR5A1, NR5A2 and SOX2 were transfected into 293T cells. Unexpectedly, *NR0B1* expression was not altered by the over-expression of AR, NR5A1, NR5A2 or SOX2 individually, or by the over-expression of all 4 factors together in the same cells (Supplementary Figure S5A-S5B).

Furthermore, a ChIP-qPCR assay showed that acetylated histone H4 binding to the promoter region of the *NR0B1* gene was enriched in 293T cells after treatment with TSA (Figure 5D), whereas the amount of acetylated histone H3 binding to the *NR0B1* promoter sequence was not significantly changed. Thus, the enrichment of acetyl-histone H4 binding to the *NR0B1* promoter region is likely to be one mechanism that facilitates its expression in 293T cells.

### Histone acetylation synergistically up-regulates *NR0B*1 gene expression on top of DNA demethylation *in vitro*

**Figure 6:**
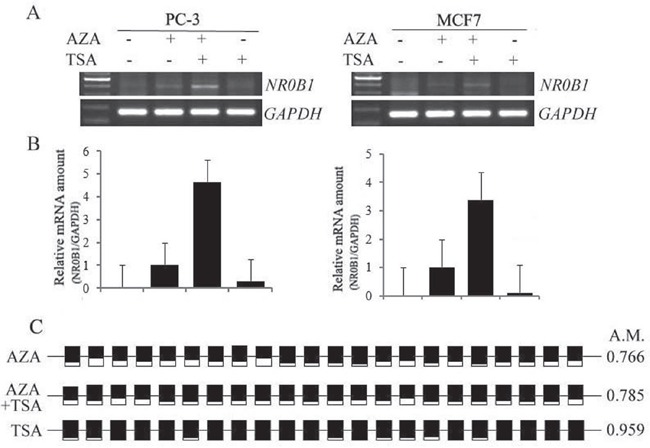
Synergistic effects of AZA and TSA on *NR0B1* gene activation **A-B.** TSA further increased *NR0B1* expression in PC-3 and MCF7 cells to a higher level than treatment with AZA alone. Treatment with TSA alone did not activate *NR0B1* expression. **C.** The DNA methylation level of the *NR0B1* CGI in PC-3 cells that were treated with AZA, TSA alone, or a combination of AZA and TSA. A.M.: average ratio of methylated CpG sites.

We next investigated whether TSA can activate the expression of the *NR0B1* gene in human cells with a hypermethylated *NR0B1* CGI. AZA and TSA have been previously reported to act synergistically during the activation of cancer-related genes [16, 17], and TSA can induce the DNA demethylation of some genes [18, 19]. PC-3 and MCF7 cells were treated with TSA alone or in combination with AZA. As shown in Figure 6A–6B, TSA treatment alone did not induce *NR0B1* expression, whereas the combination of AZA and TSA resulted in 4.5- and 3.5-fold higher *NR0B1* expression in PC-3 and MCF7 cells, respectively, than was produced by treatment with AZA alone. Subsequent bisulfate sequencing of PC-3 cells demonstrated that the methylation status of the *NR0B1* CGI was not changed by treatment with TSA. In addition, its methylation levels were decreased following treatment with AZA alone and when cells were treated with a combination of AZA and TSA (Figure 6C). However, the demethylation level observed in PC-3 cells that were exposed to both AZA and TSA was not different from the level observed in cells treated with AZA alone (Figure 6C). These results indicate that the higher fold change that was observed in *NR0B1* expression following combined treatment with AZA and TSA could be the result of synergistic action between histone acetylation and DNA demethylation in cells containing a hypermethylated *NR0B1* promoter.

### Dimethylated histone H3 lysine 9 (H3K9me2) and trimethylated histone H3 lysine 27(H3K27me3) binding to the *NR0B1* promoter are involved in silencing its expression

**Figure 7:**
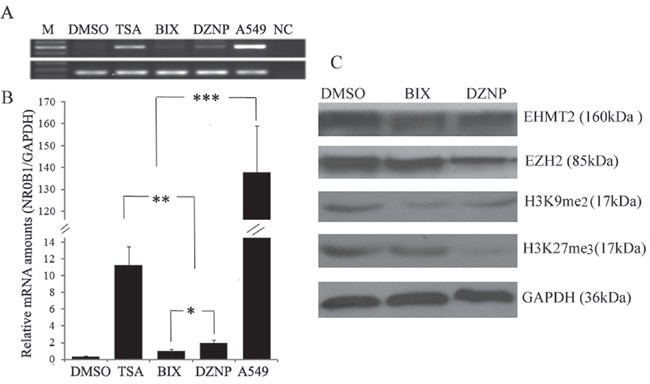
Effect of histone demethylation on *NR0B1* expression **A-B.** Treatment with the histone methylase inhibitors DZNeP (DZNP) and BIX01294 (BIX) resulted in slightly higher levels of *NR0B1* expression in 293T cells than was induced by treatment with the vehicle (DMSO). A549 acted as a positive control and water was used as a negative control (NC) in the RT-PCR analysis. **C.** The protein levels of the DZNep target methylase EZH2, the BIX01294 target methylase EHMT2, and H3K9me2 and H3K27me3 were detected in 293T cells that were treated with DZNeP, BIX01294 or vehicle (DMSO). * *p* value <0.05, ** *p* value <0.01 and *** *p* value <0.001 using the Student's *t*-test.

We next investigated the role of histone lysine methylation on the expression of the *NR0B1* gene because histone lysine methylation also plays important roles in the organization of chromatin domains and the regulation of gene expression [20]. We treated 293T cells with two histone methyltransferase (HMTase) inhibitors: 3-deazaneplanocin A (DZNep), which selectively inhibits the trimethylation of lysine 27 on histone H3 [21], and BIX01294, which selectively impairs the G9a HMTase EHMT2 and the generation of methylated H3K9 [22]. Both DZNep and BIX01294 induced the expression of the *NR0B1* gene (Figure 7A–7B), although the level of induction was lower than that observed following TSA treatment. In addition, the HMTases EZH2 and EHMT2 were depleted in 293T cells after treatment with DZNep and BIX01294, respectively (Figure 7C). Furthermore, the levels of H3K27me3 and H3K9me2 were decreased in DZNep-treated cells, whereas only the H3K9me2 level was decreased in BIX01294-treated cells (Figure 7C). These results confirm that a higher level of *NR0B1* expression is induced by treatment with DZNep than with BIX01294 (Figure 7A–7B). These results also indicate that the methylation of H3K9 and H3K27 is involved in silencing the transcription of *NR0B1* in 293T cells and *vice versa*.

### Histone modifications facilitates *NR0B1* gene expression

**Figure 8:**
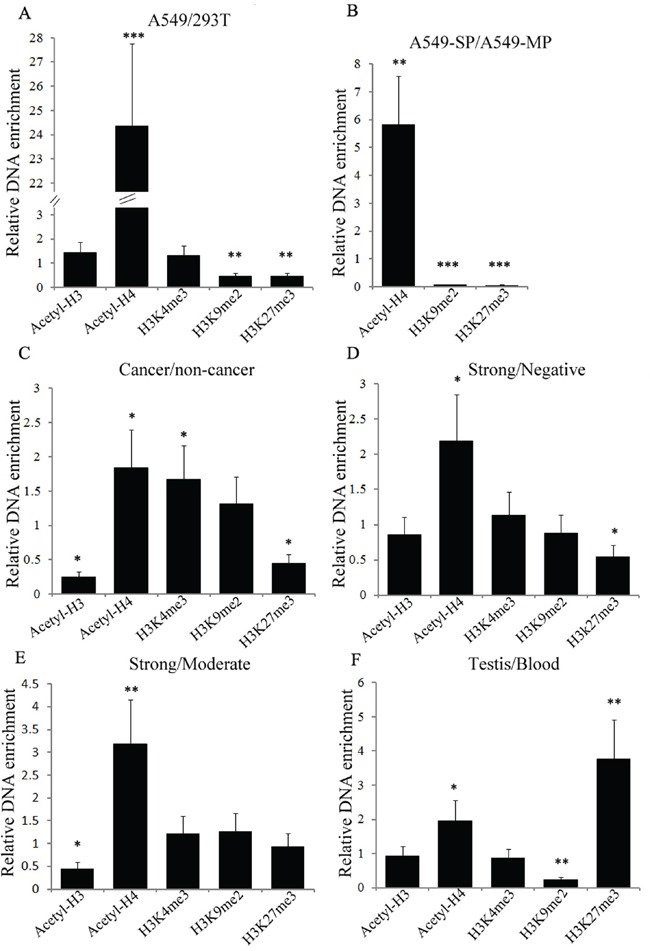
Histone modifications within the *NR0B1* promoter in different tissues and cells by ChIP-qPCR analysis Different histone modifications were detected in the *NR0B1* promoter region between 293T and A549 cells **A.** the side (SP) and main (MP) populations of A549 cells **B.** cancerous and non-cancerous tissues **C.** cancerous tissues with a strong and negative NR0B1 signal **D.** cancerous tissues with a strong and moderate NR0B1 signal **E.** and between testis tissues and peripheral blood **F.** The cancerous and non-cancerous tissues were mixed samples from 5 patients. * *p*<0.05, ** *p* value <0.01 and *** *p* value <0.001 using the Student's *t*-test.

To obtain a more comprehensive understanding of the histone modifications that promote *NR0B1* expression, we next compared the status of acetyl-histones H3 and H4 and methylated histones H3K4me3, H3K9me2 and H3K27me3 binding to the *NR0B1* gene promoter in human tissues and cells with different *NR0B1* expression patterns. The ChIP-qPCR results showed that the level of acetyl-H4 binding to the *NR0B1* promoter in A549 cells with *NR0B1* expression was more than twenty-fold higher than the level in 293T cells without *NR0B1* expression, whereas the occupancy of H3K9me2 and H3K27me3 were two-fold lower in A549 cells than in 293T cells (Figure 8A). Because the expression level of *NR0B1* was much higher in A549-SP cells than in A549-MP cells [7, Supplementary Figure S6], we compared acetyl-histone H4, H3K9me2 and H3K27me3 binding to the *NR0B1* promoter between A549-SP and A549-MP cells after both populations were sorted (Supplementary Figure S6). The results also showed that acetyl-H4 binding to the *NR0B1* promoter was approximately six-fold higher in A549-SP cells than in A549-MP cells, whereas H3K9me2 and H3K27me3 binding to the *NR0B1* promoter was minimal (Figure 8B). These results further confirm that acetyl-histone H4 binding to the *NR0B1* promoter can promote *NR0B1* expression, whereas H3K9me2 and H3K27me3 occupancy prevents *NR0B1* expression in cultured cells.

We additionally examined the above histone modifications in human tissues and cells. Consistent with the results obtained in cultured cells, the occupancy of acetyl-histone H4 and H3K4me3, which facilitate gene expression, was higher in cancerous tissues than in non-cancerous tissues, whereas the occupancy of H3K27me3 was lower in cancerous tissues (Figure 8C). Meanwhile, the acetyl-histone H4 occupancy level was also significantly higher in cancerous tissues with a strong NR0B1 signal than in those with negative or moderate NR0B1 expression (Figure 8D–8E). Histone modifications between blood cells and testis tissues also showed increased acetyl-histone H4 binding, and reduced H3K9me2 binding to the *NR0B1* promoter was observed in testis tissues (Figure 8F). Hence, the above *in vivo* results were in agreement with those obtained *in vitro*, demonstrating that hyperacetylated histone H4 and demethylated histone H3 lysine 9 and 27 binding to the *NR0B1* promoter are necessary for the activation of *NR0B1* expression.

## DISCUSSION

The orphan nuclear receptor NR0B1 is highly conserved in evolution from fish to mammals [23]. A large body of evidence obtained from experiments using animal models indicates that NR0B1 is involved in embryonic development and plays a role in the pluripotency and self-renewal capacity of embryonic stem (ES) cells. Self-renewal is an essential property of stem cells that allows them to maintain a stem cell pool. The expression of NR0B1 is regulated by STAT3, OCT3/4, NANOG, NR5A2 and estrogen receptor β (ERβ), all of which are essential transcription factors for ES cell self-renewal [14, 15, 24–26]. Recently, Fujii *et al* reported that NR0B1 is necessary for ES cell self-renewal because it represses the 2-cell-stage-specific protein Zscan4c [27]. In this study, the presence of NR0B1 was correlated with clinical stage in male ADCA. A high level of NR0B1 protein was detected in poorly differentiated male cases. Considering a higher level of expression of NR0B1 observed in SP- than in MP-derived A549 cells, we propose that NR0B1 may be involved in the process of maintaining the “stem characters” of ADCA cells and promoting self-renewal in cancer cells.

NR0B1 can act as a negative co-regulator of steroidogenic factor 1, liver receptor homolog-1, AR, ER, and progesterone receptor (PR), and it plays a critical role in the biosynthesis of steroid hormones [28]. Gonadal steroids contribute to prostate, breast, and ovary carcinogenesis [29–31], among others. The plausibility of sex hormone involvement in ADCA is also supported by the significantly higher expression of ER and PR that were observed in ADCA than in other lung cancer cell types [32–34]. In this study, the NR0B1 signal was detected in more than 50% of the cases of ADCA, indicating that the activation of NR0B1 in lung adeno-cells can promote carcinogenesis by turning on the above-mentioned signal transduction systems, including steroid hormones. Unfortunately, the levels of AR, ER and PR were not examined in this study. Further investigation into the correlations between NR0B1 and the above steroid hormone receptors will help to clarify their role in the tumorigenesis of ADCA.

In this study, more NR0B1-positive cases were observed in male than in female ADCA cases. This novel result has not previously been reported. The differential expression of NR0B1 by gender conforms to the gender difference that has been observed in the prevalence of lung cancer, even when the increase of female lung cancer prevalence is taken into account. Thus, we hypothesized that in males, NR0B1 is more easily activated than in females because of its haploid (hemizygote) state. The *in vitro* data from AZA and TSA treatment experiments indicate that NR0B1 expression can be easily induced in cells derived from males, such as PC-3, 293T and GC-1 cells.

The hypomethylation of genomic DNA is associated with the activation of CG-X genes with rich CpG residues in their promoter regions during the development of cancer [35]. Although a correlation between DNA methylation and *NR0B1* expression was previously suggested in ADCA patients, the gender of the cases that were used for bisulfite sequencing was not shown, and only 6 clones per case were sequenced [7]. However, in the current study, the sequences of more than 20 clones per sample were analyzed. Our results indicate that the CpG sites within the *NR0B1* promoter were almost unmethylated in the tissues and cells obtained from males, whereas fewer than 50% were unmethylated in the tissues and cells obtained from females, independent of the expression status of the *NR0B1* gene. In particular, no significant difference was observed in the CpG methylation level of the *NR0B1* promoter in the tissues obtained from the same gender. This methylation pattern has reserved during evolution, as revealed by investigations of the mouse *Nr0b1* CGI in multiple tissues and cells. These data indicate that DNA methylation may not be a triggering factor in the regulation of *NR0B1* expression *in vivo*. However, in cultured cells, *NR0B1* expression was silent once its CGI became hypermethylated. Thus, we propose that the unmethylated status of *NR0B1* CGIs may be necessary, but not sufficient, to induce the transcriptional activation of the *NR0B1* gene.

Acetylated histones enhance chromatin decondensation and DNA accessibility, inducing transcriptional activation. In this study, hypomethylated *NR0B1* CGIs alone were not sufficient to induce its transcriptional activation, but treatment with the HDAC inhibitor TSA induced the expression of *NR0B1* in cells with hypomethylated *NR0B1* CGIs, and treatment with TSA further promoted *NR0B1* expression higher than DNA demethylation with AZA treatment in cells with hypermethylated *NR0B1* CGIs. These observations indicate that histone acetylation status plays an important role in regulating *NR0B1* expression. When the *NR0B1* promoter contains a low level of acetyl-histone H4, its transcription cannot be activated by over-expressing the transcriptional factors AR, NR5A1, NR5A2 and SOX2 because they cannot be recruited to the binding sites on the *NR0B1* promoter. Our ChIP-qPCR results further confirmed that the acetyl-histone H4 occupancy level was correlated with the expression pattern of the *NR0B1* gene in all of the studied tissues and cells. Histone H4 is typically acetylated at lysines 5, 8, 12, and 16 [36], and the acetylated histone H4 can prevent its lysine 20 trimethylation, resulting in transcriptionally active euchromatin [37]. Moreover, the structure of acetylated histone H4 indicates that it can be recognized by the Bromodomain-PHD finger module of the human transcriptional co-activator CBP [38], which is involved in a signaling pathway during lung tumorigenesis [39–42]. Thus, we propose that hyperacetylated histone H4, in cooperation with CBP, promotes the transcription of *NR0B1* in ADCA.

**Figure 9:**
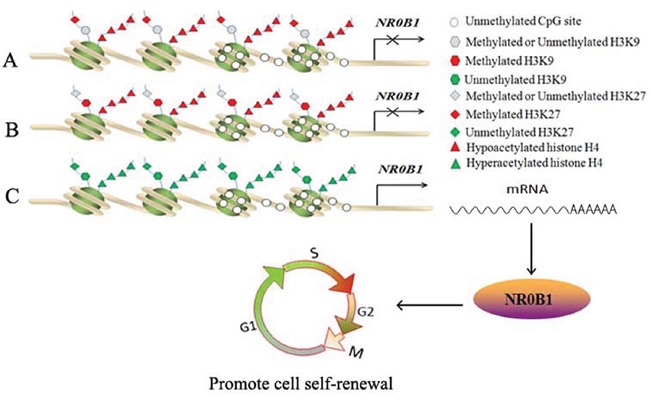
Proposed model for the *in vivo* epigenetic regulation of *NR0B1* expression **A-B.** Hypoacetylation of histone H4 and H3K9me2 (A) / H3K27me3 (B) binding to the *NR0B1* promoter silences *NR0B1* transcription, although the *NR0B1* CGI is unmethylated. **C.** In addition to unmethylated the *NR0B1* CGIs, hyperacetylated histone H4 and the demethylated lysines 9 and 27 of histone H3 on the *NR0B1* promoter facilitate its activation to promote cell self-renewal.

Methylated H3K27 and H3K9 are considered hallmark signatures of condensed heterochromatin that down-regulates neighboring gene expression [43]. In this study, treatment with HMTase inhibitors activated the expression of *NR0B1* in 293T cells. ChIP-qPCR results revealed high levels of H3K9me2 and H3K27me3 at the promoter region of the *NR0B1* gene in 293T and A549-MP cells, in which *NR0B1* was expressed at low levels, whereas the *NR0B1* CGI was hypomethylated. This result suggests that H3K9me2 or H3K27me3 binding to the *NR0B1* promoter is critical for inhibiting its transcription. Additionally, a higher level of H3K27me3 binding to the *NR0B1* promoter was found only in non-cancerous tissues and cancer tissues with negative *NR0B1* expression and not in cancer tissues with a strong NR0B1 signal. Higher H3K9me2 occupancy was observed only in peripheral leukocytes and not in testis tissues. This result suggests that the methylation of both lysines 9 and 27 in histone H3 is not required to block *NR0B1* expression *in vivo*. In contrast, either H3K9me2 or H3K27me3 may participate in inhibiting *NR0B1* gene expression *in vivo* (Figure 9A–9B).

In summary, this study demonstrates a number of epigenetic regulatory mechanisms that affect *NR0B1* expression (Figure 9). *In vivo*, we found that DNA methylation was not involved in regulating *NR0B1* transcription, although the hypermethylation of the *NR0B1* CGI silenced its expression *in vitro*. In general, the hypoacetylation of histone H4 and H3K9me2 or H3K27me3 binding to the *NR0B1* promoter silenced its transcription in normal tissues and cells. Once the hyper acetyl-histone H4 and the unmethylated histones H3K9 and H3K27 became enriched in the *NR0B1* promoter region, its transcription was activated (Figure 9C). Thus, in ADCA, the activation of NR0B1 may promote the self-renewal of cancer cells.

## MATERIALS AND METHODS

### Human samples

One hundred and sixty cancer tissues and paired adjacent noncancerous tissues (male: 83 cases; female: 77 cases) were obtained from ADCA patients who obtained a histopathologic diagnosis from 2007-2010 at the Department of Pathology, West China Hospital, Sichuan University, China. The clinical stages of the ADCA patients, including stage I (5 cases), stage II (115 cases) and stage III (40 cases), were confirmed using pathology. The ages of the ADCA patients ranged from 41 to 76 years old. Using the Decision Tree add-on module in the statistical package SPSS 17.0, we divided the ADCA patients into two groups (≤65 and > 65 years old). No correlation was observed between age and gender, between age and clinical stage or between age and NR0B1 expression level (Table S1). Human testicular biopsies were obtained from two obstructive azoospermic patients with normal spermatogenesis. Peripheral blood samples were collected from 10 volunteers (5 males and 5 females). Written informed consent was obtained from each participant.

### Mouse samples

All mouse samples were obtained from wild-type C57BL/6J mice that were bred in the Laboratory Animal Center of the State Key Laboratory of Biotherapy, Sichuan University. This study was authorized by the Ethical Committee of West China Hospital, Sichuan University.

### Cell lines

The human cell lines, including 293T (embryo kidney cell), A549 (ADCA cell), HepG2 (hepatocellular carcinoma cell), LNCaP, PC-3 and DU145 (prostate cancer cells), and the SKOV-3 (oophoroma cells), MCF7 (breast cancer cell), and HeLa (cervical cancer cell) cells were originally purchased from the American Type Culture Collection (ATCC, Manassas VA, USA) and maintained in our laboratory until used in the current study.

### Antibodies

Rabbit polyclonal antibodies for NR0B1 (ab60144), NR5A1 (ab65815), and acetyl-histone H3 (ab47915); rabbit monoclonal antibodies for NR5A2 (ab125034), NANOG (ab109250), OCT3/4 (ab109183), AR (ab133273), SOX2 (ab92494), and STAT3 (ab68153); and mouse monoclonal antibodies for H3K4me3 (ab6000), H3K9me2 (ab1220), H3K27me3 (ab6002), α-TUBULIN (ab7750) and glyceraldehyde-3-phosphate dehydrogenase (GAPDH, ab9482) were obtained from Abcam (Cambridge, MA). The rabbit polyclonal antibody for acetyl-histone H4 (06-598) was purchased from Millipore (Temecula, CA). The mouse monoclonal anti-FLAG antibody (F1804) was purchased from Sigma–Aldrich (St. Louis, MO).

### Immunohistochemical (IHC) analysis

To detect the NR0B1 protein in ADCA tissues, 5-μm sections of paraffin-embedded tissues were deparaffinized, rehydrated, and treated with hydrogen peroxide. After antigen retrieval was performed using citrate buffer and microwave boiling, the sections were incubated with anti-NR0B1 rabbit polyclonal antibodies at a dilution of 1:200 at 4°C overnight. Sections were then washed in PBS and incubated in horseradish-peroxidase–conjugated goat anti-rabbit IgG followed by incubation with 3, 3′-diaminobenzidine (DAB) substrate and counterstaining with hematoxylin solution. Pre-immune rabbit serum was used as the primary antibody for the negative controls. The level of NR0B1 staining was scored by two independent examiners and classified into three subgroups: (i) negative (−), when the NR0B1 signal was almost undetectable and the number of cells displaying NR0B1 signal was less than 1%; (ii) moderate (+), when the NR0B1 signal was moderate and the number of cells displaying NR0B1 signal was 1-50%; and (iii) strong (++), when the NR0B1 signal was strong and the number of cells displaying NR0B1 signal was greater than 50%.

### Immunoblotting (IB) analysis

Lysates that were extracted from tissues and cells were separated on 10% SDS–polyacrylamide gels and then transferred onto polyvinylidene difluoride (PVDF) membrane (Millipore). After blocking the membranes in 10% dry milk, the transferred membranes were sequentially incubated with primary antibodies and horseradish peroxidase (HRP)-conjugated secondary antibodies. Immunoreactive bands were identified using a chemiluminescent HRP substrate kit (Millipore). α-TUBULIN and GAPDH were used as the internal references. The relative NR0B1 protein levels (vs. GAPDH or TUBULIN) were calculated using Image J software.

### Bisulfite genomic DNA sequencing

Genomic DNA was extracted from tissues and cells using a Tissue DNA Kit (BioTeke Corporation, Beijing, China), and bisulfite treatment was performed using a DNA Methylation-Gold Kit (Zymo research, Irvine, CA) according to the manufacturer's instructions. The primers used for polymerase chain reaction (PCR) are shown in supplementary Table S2. All PCR products were separated on agarose gels, recovered and inserted into a T-vector (TaKaRa Bio, Dalian). More than 20 clones for each DNA sample were sequenced using T7/M13 universal primers.

### Chemical treatments

In the experiments designed to determine the effect of epigenetic factors on the expression of the *NR0B1* gene, cells were treated separately or simultaneously with the DNA methyltransferase inhibitor 5′-aza-2′-deoxycytidine (AZA, Sigma–Aldrich), the HDAC inhibitor trichostatin A (TSA, Sigma-Aldrich), and the HMTase inhibitors DZNep (Selleckchem) and BIX01294 (Selleckchem), as previously described [44, 22].

### RNA isolation and quantitative reverse transcription PCR (qRT-PCR)

Total RNA was isolated from cell lines and tissues using a Super-Purity RNA Exaction Kit (BioTeke). First-strand cDNA was reverse transcribed using a RevertAid First-Strand cDNA Synthesis Kit (ThermoFisher, Waltham, MA). The primers that were used for RT-PCR and qRT-PCR are listed in supplementary Table S2. The *GAPDH* gene was used as an internal control.

Quantitative RT-PCR was performed using the SYBR Premix Ex Taq II (TaKaRa Bio). Reactions were run using a Bio-Rad iCycler RT-PCR Detection System. The ΔΔCT method was used for data analysis. Each assay was performed in triplicate using each sample.

### Isolation of a side population (SP) from A549 cells

The SP and main population (MP) of A549 cells were isolated using fluorescence activated cell sorting (FACS, Navios MoFlo XDP, Beckman Coulter, Fullerton, CA) with Hoechst 33342 dye according to previous reports [7, 45].

### Chromatin immunoprecipitation (ChIP) and quantitative PCR (qPCR)

ChIP assays were performed using a ChIP-IT Express Enzymatic Kit (Active Motif, Carlsbad, CA). Briefly, the cells were first fixed with formaldehyde, and then the cross-linked chromatin was digested using an enzymatic shearing cocktail. A portion of optimally sheared chromatin was kept for use as a control “input DNA” in the subsequent qPCR analysis. The remaining sheared chromatin was precipitated by incubation with antibodies against acetyl-histone H3, acetyl-histone H4, H3K4me3, H3K9me2 and H3K27me3. qPCR was performed to amplify the target region of the *NR0B1* promoter. The CT values from each run were averaged per tissue or cell, and the ΔΔCT method was then used for the data analysis, in which the value of target DNA fragments that were enriched in each tissue or cell was normalized to the value of the 5% input DNA of each sample. The qPCR data were expressed as the means ± standard deviations (S.D.). The ChIP-qPCR assay was repeated twice to confirm the reproducibility of the results.

### Statistical analysis

Pearson's chi-squared tests were used to calculate the differences in the expression of NR0B1 between different genders and clinical stages. Student's *t*-test was used in all other comparisons. A *p* value of less than 0.05 was considered significant. R program (version 3.2.2) and GraphPad Prism 5 software were used for the statistical analysis.

## SUPPLEMENTARY MATERIAL FIGURES AND TABLES


