# Supplementary material for: Epigenetic modifications promote the expression of the orphan nuclear receptor NR0B1 in human lung adenocarcinoma cells
Source: Oncotarget. 2016 Apr 26;7(28):43162–76. doi: 10.18632/oncotarget.9012 (PMC5190015; doi:10.18632/oncotarget.9012)
Supplement: Supplementary file 1 [file oncotarget-07-43162-s001.pdf]

Epigenetic modifications promote the expression of the orphan nuclear receptor NR0B1 in human lung adenocarcinoma cells

SUPPLEMENTARY MATERIALS AND METHODS

Plasmid construction

A CpG-free luciferase reporter vector without a promoter (pCpGf-basic) and a CpG-free luciferase reporter vector with a human elongation factor 1 alpha core promoter (pCpGf-luc) were constructed. The promoter containing the CpG island (CGI) region of human *NR0B1* was amplified and inserted into the pCpGf-basic vector to produce the pCpGf-NR0B1pro vector. Similarly, the homologous region of the mouse *Nr0b1* gene was obtained to construct the pCpGf-Nr0b1pro vector. All of the constructs were verified using sequencing. The plasmids used to induce the expression of the transcription factors AR, NR5A1, NR5A2 and SOX2 were purchased from GeneCopoeia (Rockville, MD).

In vitro methylation assays

The *NR0B1/Nr0b1* promoter construct DNA was treated with CpG methyltransferase (M. SssI) (New England Biolabs (NEB), Hitchin, UK) according to the manufacturer's recommendations in the presence of S-adenosylmethionine (NEB). Its methylation efficiency was evaluated by digestion with the methylation-sensitive restriction enzyme HpaII (NEB). DNA purification was performed using the phenol/chloroform method.

The methylated and unmethylated constructs were transfected separately into 293T and B16 cells using a jetPRIME transfection kit (Polyplus, Illkirch, France) according to the manufacturer's instructions. The pRL-CMV vector (Promega, Madison, WI) was cotransfected to normalize transfection efficiency. After 48 hours, the luciferase activity of the cell lysates was analyzed using a dual-luciferase reporter assay system (Promega).

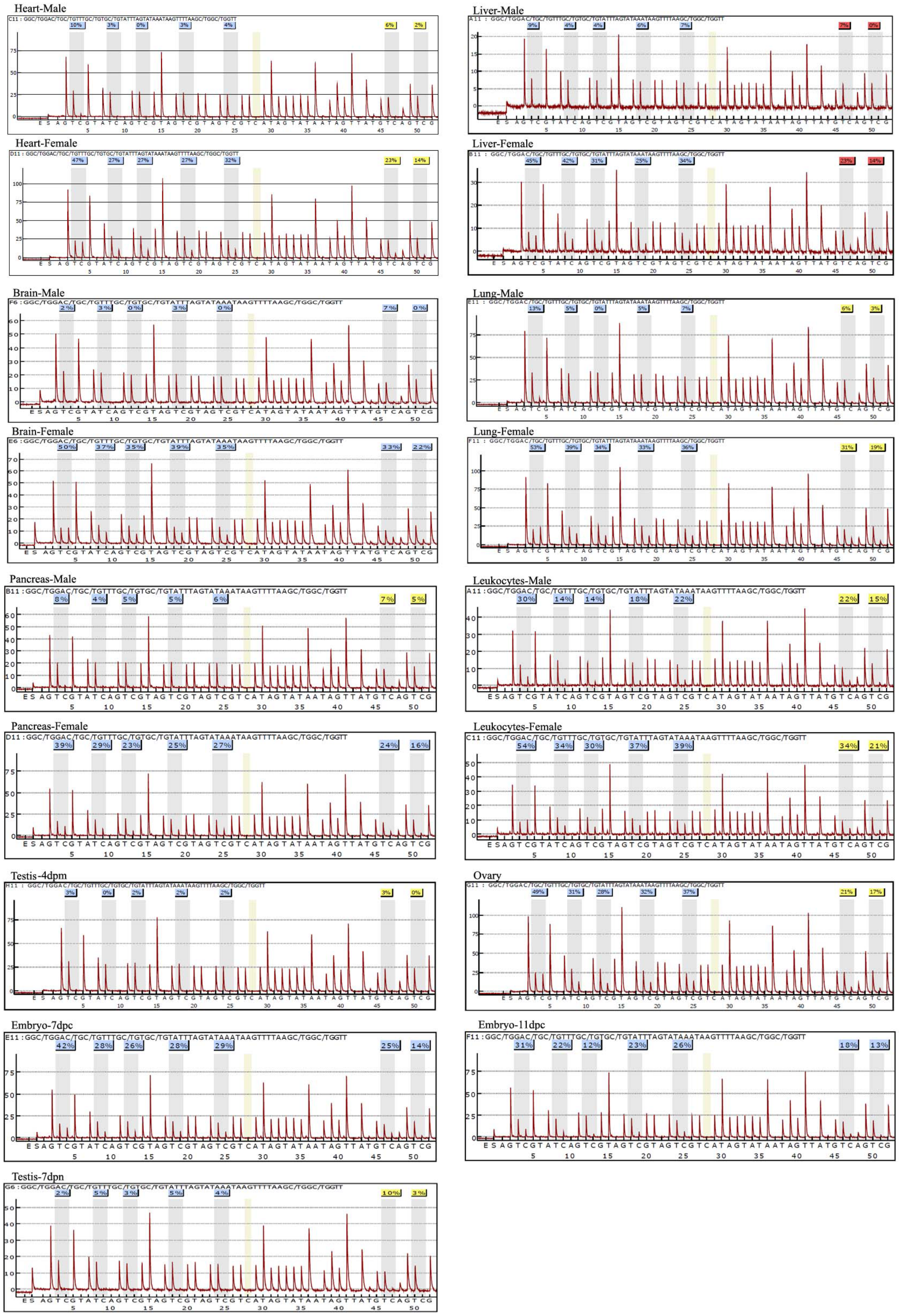

Supplementary Figure S1: The CGI methylation pattern of the *Nr0b1* gene in mouse tissues and cells was determined using pyrosequencing. The CpG sites were hypomethylated in all of the tissues and cells obtained from male mice. Fewer than 40% of the CpG sites in the parallel tissues and the cells obtained from female mice were methylated.

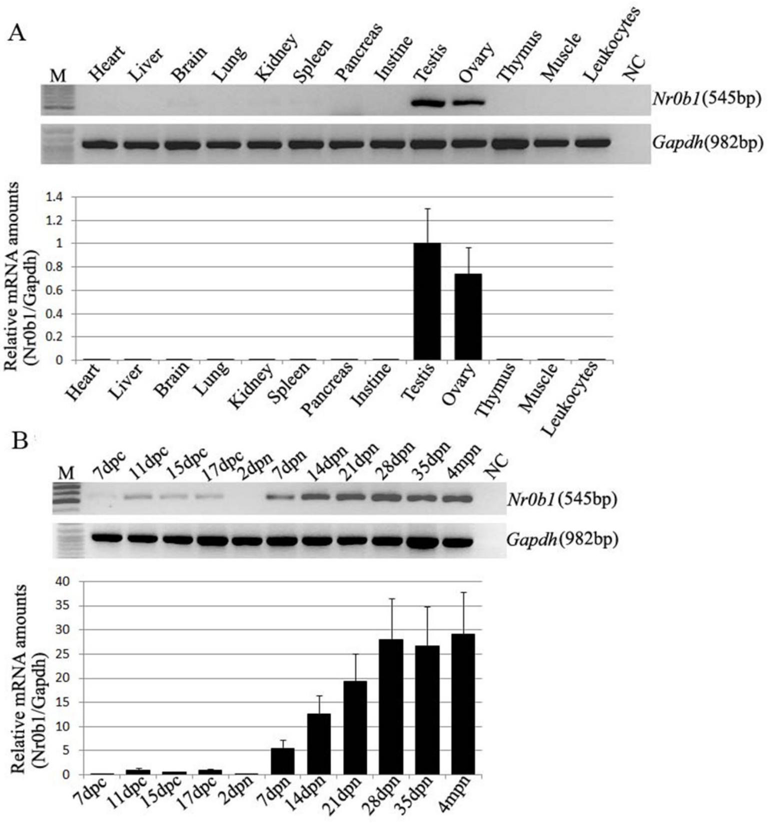

Supplementary Figure S2: Expression pattern of the mouse *Nr0b1* gene in multiple tissues and cells. A. Mouse *Nr0b1* gene expression was restricted to the testis and ovarian tissues (upper panel: gel electrophoresis results from RT-PCR; lower panel: qRT-PCR results). **B.** The mouse *Nr0b1* gene was expressed at a low level in embryos and was first expressed in the testis at 7 days post-natal (dpn); upper panel: gel electrophoresis results from RT-PCR; and lower panel: qRT-PCR results. dpc: days post-coitus, mpn: months post-natal. NC: Water was used as a negative control in the RT-PCR analysis.

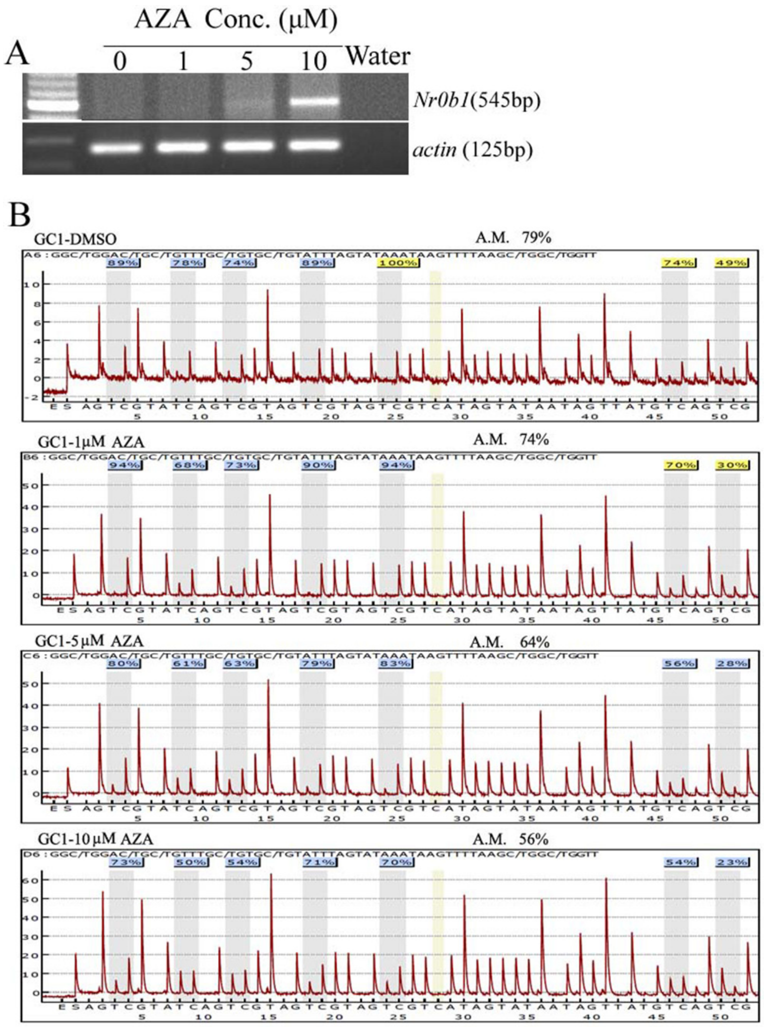

Supplementary Figure S3: DNA demethylation activated mouse *Nr0b1* gene expression in GC-1 cells. A. *Nr0b1* expression was activated in GC-1 cells by treatment with the DNA methyltransferase inhibitor AZA. **B.** Pyrosequencing showed that the CpG sites in the promoter region of the *Nr0b1* gene were partially demethylated after AZA treatment.

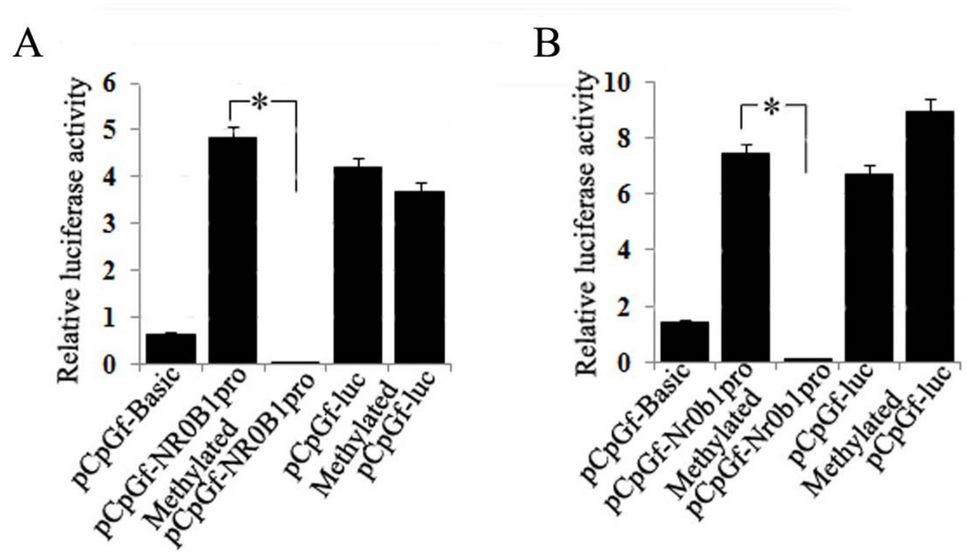

Supplementary Figure S4: Luciferase activity resulting from the methylated and unmethylated human *NR0B1*. A. and mouse *Nr0b1* **B.** promoter constructs in 293T and B-16 cells, respectively. * p value <0.05 according to the Student's t-test.

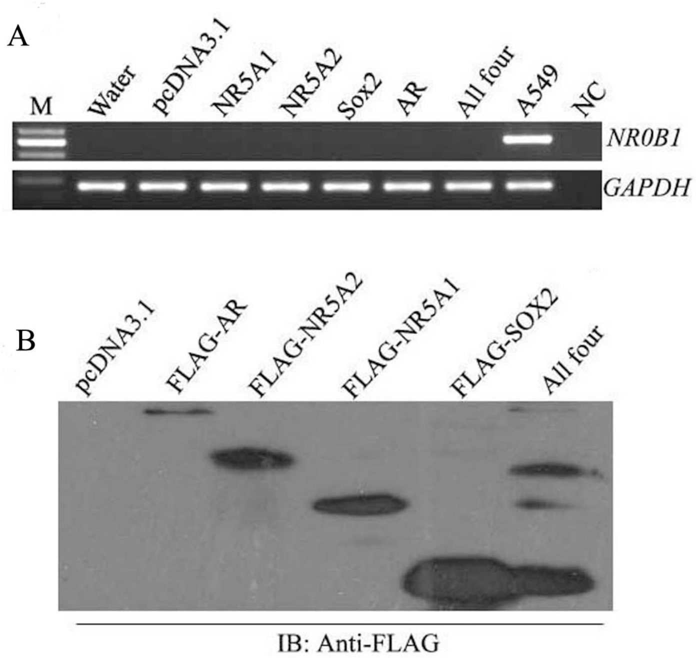

Supplementary Figure S5: A. *NR0B1* expression was not activated in 293T cells by over-expressing AR, NR5A1, NR5A2 and SOX2 individually or in combination. **B.** Immunoblotting (IB) analysis was performed using an anti-FLAG antibody on cell lysates from 293T cells that over-expressed AR, NR5A1, NR5A2 and SOX2 individually or in combination.

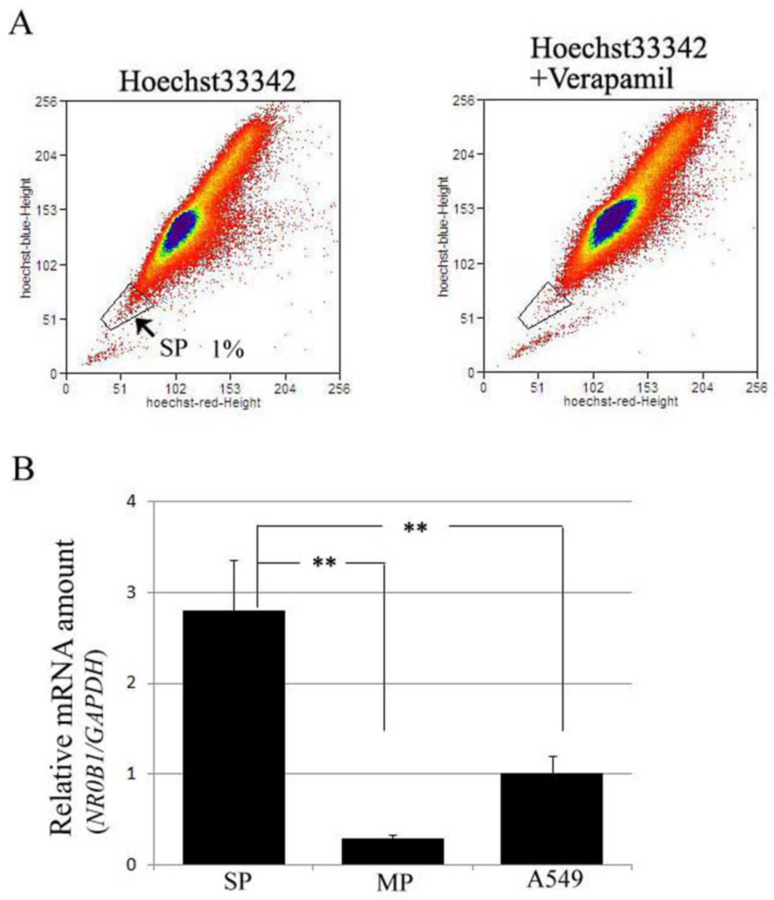

Supplementary Figure S6: Isolation of A549-SP cells by fluorescence-activated cell sorting (FACS) using Hoechst 33342 dye. A. The FACS results showed that SP cells accounted for approximately 1% of the total number of A549 cells. **B.** The relative level of *NR0B1* expression in A549-SP, -MP and total A549 cells. The expression level of *NR0B1* was significantly higher in A549-SP cells than in A549-MP and total A549 cells. ** p value <0.01 according to the Student's t-test.

Supplementary Table S1: The correlation of age with gender, clinical stage and NR0B1 expression level in the human lung adenocarcinoma cases

		Age [a]		*p* value (α=0.05)[b]
		**≤65 (104 cases)**	**>65 (56 cases)**	
**Gender**	Male	51	32	0.249
	Female	53	24	
**Clinical Stage**	I	3	2	0.972
	II	75	40	
	III	26	14	
**NR0B1 signal level**	Strong (++)	24	21	0.118
	Moderate (+)	31	11	
	Negative (-)	49	24	

[a] The Decision Tree add-on module in the statistical package SPSS 17.0 suggested that the variable age of lung adenocarcinoma patients could be divided into two groups (≤65 and > 65 years old).
[b] Pearson's chi-squared test is applied in the calculation.

Supplementary Table S2: Primers used in this study

Primer Name	Primer Sequence	Product Size (bp)	Application
HuNR0B1F	ACCACCAGGCGGCGGGAGACC	398	RT-PCR for amplifying human *NR0B1* gene
HuNR0B1R	CCGATGATGGGCCTGAAGAACA		
MmNR0B1F	GGAGATCCCGGAGACCAACAC	545	RT-PCR for amplifying mouse *Nr0b1* gene
MmNR0B1R	GCTACGACCGGCTTTCTCCATCTA		
GAPDH-f1	TGAAGGTCGGAGTCAACGGATTTGGT	982	An internal control for RT-PCR
GAPDH-r1	CATGTGGGCCATGAGGTCCACCAC		
DAX-IF	CTACCTCAAGGGGACCGTGCT	237	qRT-PCR for human *NR0B1* gene
DAX-IR	ATGCTGACTGTGCCGATGATG		
MDAX-RTf	AAGTGCTGGAGTCTGAACATTG	238	qRT-PCR for mouse *Nr0b1* gene
MDAX-RTr	CAGTGACGACATCGCTATTGA		
GAPDH-f2	AGCCGCATCTTCTTTTGC	163	An internal control for qRT-PCR
GAPDH-r2	AATGAAGGGGTCATTGATGG		
HuNR-BSPf	GAAGGAGGAAAGTGTTTAGGAGTTT	238	Bisulfite sequencing
HuNR-BSPr	AACCCAATTCTACCCAATAACTACC		
HuNRpf	ACTCGAGGGCATTTCCACCGAGTTC	528	Human *NR0B1* promoter construction
HuNRpr	CAAGCTTGCGCCCGTAGCCCAGTT		
MmNrpf	ACTCGAGCATTTGTGCAGCCTGGGTGTGA	587	Mouse *Nr0b1* promoter construction
MmNrpr	CAAGCTTGGCCGCCGCTTGGGACTTA		
NR-ChIPf1	CCTGAGACAGGGAAAGGGGT	112 (primer 1)	ChIP-qPCR for *NR0B1* gene promoter region
NR-ChIPr1	TGTGTTCGCCCATGACCTC		
NR-ChIPf2	CTGGGCAGAACTGGGCTAC	126 (primer 2)	
NR-ChIPr2	CTCTGGAGCCTCAGGAGC		
